# Automated Assessment of Pain: Prospects, Progress, and a Path Forward

**DOI:** 10.1145/3461615.3485671

**Published:** 2021-12-17

**Authors:** Kenneth Prkachin, Zakia Hammal

**Affiliations:** University of Northern British Columbia; Carnegie Mellon University

**Keywords:** Pain, Nonverbal behavior, Multimodal, Self-report, Automated assessment

## Abstract

Advances in the understanding and control of pain require methods for measuring its presence, intensity, and other qualities. Shortcomings of the main method for evaluating pain—verbal report—have motivated the pursuit of other measures. Measurement of observable pain-related behaviors, such as facial expressions, has provided an alternative, but has seen limited application because available techniques are burdensome. Computer vision and machine learning techniques have been successfully applied to the assessment of painrelated facial expression, suggesting that automated assessment may be feasible. Further development is necessary before such techniques can have more widespread implementation in pain science and clinical practice. Suggestions are made for the dimensions that need to be addressed to facilitate such developments.

## 1 INTRODUCTION

Pain is an almost-universal experience for humans and other animals. The ability to feel pain is fundamentally adaptive because it motivates several different types of behavior that limit its causes, modulate its intensity, promote healing and survival, and communicate its existence and properties. Those exceptional humans who do not experience pain typically live short lives. Nevertheless, it is the prototypic source of suffering and, next to sustaining life itself, its elimination or control is the principal task of health-care. Painful conditions are responsible for high levels of morbidity, diminished quality of life, and enormous economic burden world-wide. Although some causes of pain (for example, fractures) are common and obvious, others (for example, fibromyalgia) are not and pain science increasingly demonstrates its complexity.

According to the International Association for the Study of Pain’s recently revised definition, pain is “an unpleasant sensory and emotional experience associated with, or resembling that associated with, actual or potential tissue damage” (Raja et al, 2020). Its scientific understanding and clinical control depend ultimately on the ability to infer its presence, its intensity, and other qualities such as its unpleasantness and temporal dynamics. This poses an epistemological problem since there is no way to measure the subjective experience directly. Almost all assessment of pain is based on some quantifiable feature of the sufferer’s behavior. Most often that is the sufferer’s verbal report which, for many purposes, is adequate. There is a voluminous literature devoted to testing and articulating the strengths and weaknesses of various self-reported verbal measures. Although verbal report is sometimes characterized as the “gold standard” for pain assessment, it remains problematic from a scientific perspective for numerous reasons, including its subjectivity, uncertain underlying metric properties, sometimes problematic reliability, and obtrusiveness.

Pain evaluation and management are thus complicated at the best of times. The complication is amplified in a situation in which the principal criterion on which inferences about it are made—the verbal report—is unreliable or absent as is frequently the case among neonates, young children, or patients with cognitive impairments (e.g., dementias) (Craig et al, 2011; Kunz et al., 2007). Clinicians can use other forms of evidence, such as physiological recordings, reports of staff, caretakers, or family members, all of which are problematic from the perspective of evidence of reliability, validity, and sensitivity. Evidence supports the reliability and validity of pain measurement via nonverbal behavior (facial expression) (Grunau & Craig, 1990; Prkachin and Solomon, 2008). However, human based manual observations of nonverbal behavior are impractical for many purposes. Automated assessment of pain from nonverbal behavior has emerged as a possible additional source of information for a more objective assessment and management of pain.

## 2 BEHAVIORAL ASSESSMENT OF PAIN

The recognition that inferences about pain are made on the basis of behavior has stimulated interest in the properties of other pain phenomena that can be observed directly. Some behaviors, such as withdrawal or limping, most likely originate as attempts to control it. Others, such as changes in facial expression do not appear to affect the experience directly. Although both can support inferences about pain in others, facial expressions appear to primarily serve a social communicative function. Techniques developed to manually characterize facial expressions have been extensively applied to the assessment of pain. There is now a considerable and reasonably consistent literature characterizing the properties of facial expressions during pain (Prkachin and Solomon, 2008; Rash et al., 2019). The most common assessment technique involves direct measurement of facial actions via the Facial Action Coding System (FACS) (Ekman et al, 2005). FACS is applied by highly trained human annotators who observe and then record the presence or intensity of operationally-defined Action Units (AUs). Research using FACS or similar procedures generally supports the conclusion that there is a limited set of facial actions that encode pain, they are consistent across pain arising from different modalities, and homologous across the life span (Prkachin, 2009). Those actions can be combined into an additive index that is sensitive to variations in pain experience. Under the right recording circumstances, they can be quantified unobtrusively and continuously (Prkachin and Solomon, 2008).

An alternative, though simpler, approach to assessing facial expression in pain is the judgment study. Using this technique, naïve raters view recordings of subjects who may be in pain and quantify how much pain they appear to be in by using some kind of rating scale. The number of naïve raters is adjusted to meet a target reliability criterion for averaged ratings (e.g., intraclass correlation >=0.80) (Rosenthal, 2005). The obtained aggregate intensity is then used as the ground truth of pain intensity score. In addition to their greater simplicity and reduced burden, judgment studies have further advantages over measurement of specific facial actions. They are based on a holistic analysis that does not assume independence of an expression’s component actions and probably represents human perceptual processing more realistically.

Application of knowledge about facial expressions of pain has unfortunately been limited both scientifically and clinically because it is resource-intensive. Direct observation techniques, such as FACS or derivatives, require extensive training. Their application is laborious and time-consuming. Certain potentially informative features of facial movements such as dynamic temporal changes are so laborious to obtain that few datasets contain the information. These have proven to be significant barriers to the wider adoption of standardized observational methods in clinical settings. Less resource-intensive techniques, such as judgment studies, still require considerable dedicated time of human observers in order to meet reliability requirements. Since the annotations of human observers are based on pattern recognition, it has long been hoped that techniques based on advances in computer vision and machine learning could provide an alternative that would address the problem of measurement burden. Early work on automated assessment of facial AUs and facial expressions of emotion supported the feasibility of this idea.

## 3 AUTOMATED ASSESSMENT OF PAIN

Application of computer vision and machine learning to the detection and measurement of pain requires significant collaboration between technical and clinical experts. Such collaboration is needed first for the development of datasets with an independent standard for determining that someone is in pain and a quantification of its intensity to establish ground truth. Among the first efforts in the field, Brahnam et al (2006) used still images of neonates’ responses to heel lancing compared with four other affective states. They were able to achieve 88% correct classification distinguishing pain from all other conditions with a Support Vector Machine (SVM) approach. Ashraf et al (2009) was the first effort in the field that made use of video recordings of adult participants with shoulder pain. Participants were video recorded while undergoing a series of range-of-motion exercises to their affected limbs (Prkachin & Solomon, 2008). The video recordings were rated for pain intensity at the video level by an independent observer and at the frame-byframe level using the FACS. By training a SVM to classify sequences or individual frames they were able to show acceptable classification of pain vs. no pain. In a further analysis of the Ashraf et al (2009) shoulder pain study, Lucey et al (2011) used a combination of Active Appearance Model representations with SVM’s, achieving a ROC area-under-the-curve A’ value of .84 in predicting pain as evaluated by a FACS-based pain intensity metric. Littlewort et al (2009) applied a previously developed system for detecting FACS AUs in an experimental pain setting. Participants were exposed to two conditions. In one they immersed an arm in ice water; in the other they “faked” being in pain. Automatically processed FACS AU parameters were processed using a Gaussian SVM, yielding an 88% correct classification of faked or genuine pain, substantially exceeding the comparable 49% correct performance of naïve human observers. Hammal and Kunz (2012) studied facial responses to experimental heat pain and posed expressions of several emotions, in addition to pain. FACS AUs were used to establish ground truth. Using a technique based on the Transferable Belief Model and dynamic information from the video recordings, they were able to achieve a correct classification rate of 81.1% distinguishing pain from no pain in the experimental heat arm of the study, and of 84.5% discriminating pain in an 8-alternative forced-choice task involving other expressions of emotion. Using pain data collected in different contexts, these pioneering efforts have shown that automated measurement of facial expression of pain can provide a reliable and clinically useful information for pain assessment.

A database based on Lucey et al (2011), consisting of video of participants with shoulders pain injury and related pain annotations has since been made available to qualified researchers whose work is consistent with the conditions to which the shoulder pain patients consented. Since being made available, the UNBC-McMaster Shoulder Pain Archive (Ashraf et al (2009); Lucey et al (2011)) has been employed in scores of computer vision studies (Hammal et al., 2018; Walter et al., 2019). Since being made publicly available, the UNBC-McMaster Shoulder Pain Archive has been the most widely used dataset for automatic pain detection and intensity measurement from facial expression (e.g., Ashraf et al., 2007, 2009; Hammal et al., 2012; Florea et al., 2014; Kaltwang et al., 2012; 2016; Lucey et al., 2011, 2012; Rudovic et al., 2013; Sikka et al., 2014, 2015; Liu et al. 2017; Rathee et al., 2016; Ruiz et al., 2016; Martinez et al., 2017; Werner et al., 2014, 2017; Erekat et al. 2020; Szczapa et al., 2020).

## 4 LIMITATIONS AND FUTURE DIRECTIONS FOR AUTOMATED ASSESSMENT OF PAIN

By now numerous successful applications of computer vision to assess facial expressions related to pain have established the basic feasibility of the idea to the extent that first-generation commercial products, based on earlier research are being marketed (e.g., www.painchek.com). Nevertheless, despite rapid progress in the area, there are limitations to the existing corpus that need to be addressed before contributions from this field can achieve significant scientific and clinical impact.

### 4.1 Generalizability

One major concern is the generalizability of the various automated approaches for pain measurement that have shown promise. Existing models for automatic pain assessment have been developed and tested on a limited set of databases, selected for properties that are compatible with requirements of the technology. Consequently, we cannot be comfortable that the solutions are broadly representative of pain expression. This is linked to the associated problem of algorithmic bias. For instance, in a relevant recent paper, Taati et al (2020) showed that existing facial landmark detection algorithms performed differently among independent living seniors than seniors with dementia. These results highlight the need for broader availability of datasets that sample adequately for personal characteristics known to affect the experience and perception of pain in others; namely, “race,” sex, age, and culture. For example, there is evidence for racial, ethnic, and sex differences both in how pain is expressed and how expressions are perceived by others. Most existent databases used for the development automated approaches for pain assessment do not have a broad sampling of these variables, and all have been collected in western, industrial societies. As a consequence, none of the proposed approaches for automated assessment of has investigated the possible influence of individual differences such as gender and ethnicity.

Researchers in automated assessment of pain also need to be sensitive to important distinctions among the types of pain studied that can limit the generalizability of the conclusions that they draw. Experimental pain induction has certain scientific advantages—primarily high control over stimulus and recording parameters—that come at the cost of ecological validity. Clinical pain, which can be acute or chronic and varies according to etiology, is accompanied to a greater extent by correlated affective states such as anxiety and depression and important cognitive features such as concern over its meaning, all of which can modulate the pain experience and the way that it is expressed. This is not to say that automated assessment using experimental pain is irrelevant to clinical pain. There is evidence for commonalities in the way pain is expressed across pain states (Kunz et al, 2020; Prkachin, 2009).

### 4.2 Multimodality

The focus on facial expression for pain assessment has been driven by its central importance in the study of affective states, the existence of well-accepted methods for its measurement in behavioral studies, and accumulated knowledge from programmatic studies of properties of facial expressions of pain. The existence of a rigorous method for observational measurement of facial expression has dominated the field, possibly to its detriment because its laborious nature has slowed the pace of discovery. Indeed, facial expression and verbal reports are not the only behaviors that communicate information about pain experience. For instance, body movement is an important behavioral index of pain in patients with cognitive impairments, and those who have difficulty communicating verbally (Warden et al., 2003, Arif et al., 2010; Chan et al., 2014; Fuchs-Lacelle et al., 2008; de Knegt et al., 2013; Hadjistavropoulos et al., 2018). There is a venerable tradition of observational methodologies to assess other common pain indicators, such as nonverbal vocal expressions, guarding behavior, self-soothing, and so on (Keefe et al, 2011) and progress toward technology-assisted assessment of some of these indicators (e.g., guarding) has recently been reported (Olugbade et al, 2018). Each of these modalities present its own challenge in terms of finding an effective technology to render them compatible to analysis but their incorporation into a multimodal methodology holds promise for a more accurate and sensitive measurement of pain.

### 4.3 Utility

A need for behavioral assessment of pain among people with verbal communication deficits has long been recognized (Prkachin, 2011). Automated continuous assessment in neonatal intensive care or among adults with verbal communication deficits such as many in long-term care facilities could provide a valuable tool to guide clinical intervention. Knowledge about pain expression in neonates and young children has advanced considerably and should provide an effective basis for developing and refining automated assessment of pain in these populations. Development of techniques for assessing pain in the elderly, including people with dementia is an active area of current focus Taati et al (2020).

Additionally, there is evidence that pain intensity during a clinical episode or a personal trait of pain reactivity has long-term impact on the outcome of pain conditions, disability, and quality of life. A test of the utility of automatic assessment of pain would be if it could improve the ability to predicting such clinical outcomes. As methods develop and improve, opportunities to combine automated measurement procedures with assessments of the short-, intermediate-, and long-term outcomes of pain conditions and their treatment should be sought.

### 4.4 Deployment

Automated assessment of pain has the potential to become another source of valuable information in addition to clinical intuition and self-reported pain. Such a measure could be deployed in experimental studies of pain to determine the effects of pain modulation interventions such as medications or cognitive-behavioral interventions. Such a measure could also provide a valuable tool to guide clinical intervention in neonatal intensive care or among non-communicating adults especially in long-term care facilities. From both technical and clinical perspectives, an optimally useful system for measurement of pain would be fully automated and capable of rendering information about the presence and intensity of pain on a continuous basis and in the context in which it happens (e.g., using smartphones). To achieve this goal, advancement in automatic assessment of pain cannot happen in parallel (data collection and then algorithms development). It needs close iterations from early stages of conceptualization of the technology until it’s delivery to clinical practice.

In order to be widely used in clinical practice, advanced machine learning algorithms for automatic pain measurement must be trained on a wide variety of naturalistic clinical datasets. Reliability, generalizability, and interpretability must be secured for any practical health prediction system. Given the variety of pain experiences, a variety of participants and experimental and observational procedures are needed to develop reliable computational approaches for different kinds of clinical pain (Hammal et al., 2019).

## CCS CONCEPTS

• **Applied computing** → Life and medical sciences; Health informatics;; • **Human-centered computing** → User interface design; User models;.
